# How Children Are Protected From COVID-19? A Historical, Clinical, and Pathophysiological Approach to Address COVID-19 Susceptibility

**DOI:** 10.3389/fimmu.2021.646894

**Published:** 2021-06-11

**Authors:** Magdalena Anna Massalska, Hans-Jürgen Gober

**Affiliations:** ^1^ Department of Pathophysiology and Immunology, National Institute of Geriatrics, Rheumatology, and Rehabilitation, Warsaw, Poland; ^2^ Department of Pharmacy, Kepler University Hospital, Linz, Austria

**Keywords:** COVID-19, natural IgM, children, Kawasaki-like disease, ACE-2 receptor, reactive oxygen species, silk road, B1 B cells

## Abstract

The origin and the global spread of severe acute respiratory syndrome coronavirus 2 (SARS-CoV-2) causing coronavirus disease 2019 (COVID-19) in early 2020 was accompanied by high rates of mortality in regions belonging to the ancient silk road, such as the south of China, Iran, Turkey and the northern parts of Italy. However, children seem to be spared in the epidemic as very small percentage worldwide being ill. The protection of children and neonates suggests the involvement of a specific component of adaptive immunity present at early development. Native immunoglobulin belonging to the class of IgM is abundantly present in neonates and children and is known for its recognition of self- and altered self-antigens. Native IgM may be able to neutralize virus by the recognition of endogenous “danger signal” encoded in the viral envelope and originally imprinted in the membranes of infected and stressed cells. Noteworthy, thrombosis and vasculitis, two symptoms in severely affected adult and pediatric patients are shared between COVID-19 and patients with Behcet’s disease, an autoimmune disorder exhibiting a region-specific prevalence in countries of the former silk road. Molecular mechanisms and clinical indicators suggest reactive oxygen species as trigger factor for severe progression of COVID-19 and establish a link to the innate immune defense against bacteria. The selective pressure exerted by bacterial pathogens may have shaped the genetics of inhabitants at this ancient trade route in favor of bacterial defense, to the detriment of severe COVID-19 progression in the 21th century.

## Introduction

On March 11, 2020, the World Health Organization (WHO) has declared COVID-19 as a pandemic a disease resulting from the infection by SARS-CoV-2, a novel member of the coronoviridae family (1). In severe cases, the disease manifests by interstitial pneumonia and alveolar damage within approximately 7 days of symptom onset, which can lead to acute respiratory distress syndrome (ARDS) (2, 3). ARDS is associated with an uncontrolled immune activation, characterized by a massive upregulation of pro-inflammatory cytokines, chemokines, and hematopoietic growth factors (IFN-γ, IL-1β, IL-6, IL-7, IL-8, IL-2, TNF, CXCL10, CCL2, GM-CSF) called cytokine storm (3–5), culminating in hyperinflammation and multi-organ disease (6), which is the leading cause of mortality (7). Septic shock and multiorgan failure were the most common immediate cause of death, often due to suppurative pulmonary infection as shown by Elezkurtaj et al. (8).

As of December 23, 2020, SARS-CoV-2 has been the cause of infection in more than 78 million people resulting in more than 1.7 million deaths worldwide; however, comparatively, few infections have been described in children. In contrast to infected adults, most children with confirmed SARS-CoV-2 infection seems to have a mild clinical course, and almost 16% of them did not show any symptoms of infection (9). By March 25, 2020, Italy had the second highest number of COVID-19 infections and the highest number of deaths worldwide, but only 1% of the total number of patients were children younger than 18 years of age, although children comprise 10% of the total population. Only 11% of those affected children required hospitalization, and none of them died (10).

Two distinct mechanistic checkpoints seem to determine the disease outcome following infection with SARS-CoV-2. First, the differential in host susceptibility to viral infection leading to clinical symptoms, and second, the severe deterioration of disease associated with cytokine storm and mortality. Thereby, susceptibility and mortality were found to be a matter of age and geographic location, respectively.

The immunity against COVID-19 in children and the differences in mortality between geographic regions during the initial spread of SARS-CoV-2 in the spring of 2020, prompted us to address those questions from several perspectives, encompassing immunology, virology, cell biology, historical, and linguistic sciences. The synopsis across different viewpoints culminated in two hypotheses, as proposed in this review.

First, we summarize the current knowledge on COVID-19 in children. The following two sections deal with less recognized facts on the cell biology of SARS-CoV infection and the difficulty to explain protection by an IgG-mediated mechanism directed against spike protein. Finally, we conclude our hypothesis based on a danger model of adaptive immunity. In the next two chapters, we elucidate the current knowledge on B1 B cells and natural IgM in viral defense and how these may apply to the SARS-CoV-2 pandemic.

Our two last sections are devoted to understanding the ethnic and global differences in COVID-19–related mortality. In section entitled Mortality: *Why Some Patients End Up on Ventilators and Cytokine Storm in COVID-19?*, we discuss the role of receptors, signaling proteins, and enzymes involved in the generation of reactive oxygen species and their association with the cytokine storm. In last section (*Genetics of ROS Generation and Human Sociographic and Linguistic Evolution*), we explain our hypothesis on polymorphisms associated with bacterial defense and simultaneously determine the outcome of COVID-19 disease.

## Susceptibility—Covid-19 In Children And Specifics In Pediatric Immunity

Children with SARS-Cov-2 infection present milder symptoms and show less laboratory and radiologic abnormalities compared to adults (11). The same observation was reported during the SARS and MERS-CoV epidemics in 2003 and 2012, respectively (12, 13). Depending on the study, up to 35% of pediatric patients with COVID-19 are asymptomatic. It remains uncertain whether asymptomatic children transmit the virus; however, it was shown that even asymptomatic children can have high viral loads of SARS-CoV-2 (14).

Although most of the children infected with SARS-Cov-2 present a less severe form of COVID-19, in April 2020, the UK reported a growing number of cases with features similar to atypical Kawasaki disease (15). Among the first eight hospitalized children reported, six were of Afro-Caribbean, one of Asian and Middle-Eastern descent each, and five of them were boys mostly overweight, suggesting that the ethnic characteristic prone to COVID-19 infection in children resembles the group of adults with high risk for COVID-19 mortality (16). Interestingly, no pathological organism was identified in seven from eight children during hospital treatment (15), indicating a self-preserving inflammatory disease triggered by viral exposure and persistent even after the immunologic clearance of the virus.

Recently, a novel multisystem inflammatory syndrome in children (MIS-C) belonging to the spectrum of Kawasaki disease-like pathology have been reported in regions with receding SARS-CoV-2 epidemics (17). Inflammatory shock, gastrointestinal symptoms, and coagulopathy, which are rarely seen in classic Kawasaki disease, are prominent features of this syndrome, affecting mostly older children (mean age was 12 in that study) with Black or Hispanic ethnicity. All children harbored antibodies against SARS-CoV-2 of class IgG and IgA, with absence of circulating IgM in most cases (18). Detection of autoantibodies of IgG and IgA class against endothelial, mucosal, and immune antigens together with neutrophil and monocyte upregulation of CD54 and CD64 suggest that autoreactivity together with inflammatory innate immune response may be critical to the pathogenesis of MIS-C (18). The two syndromes connected with SARS-Cov-2 infection and described in children, Kawasaki-like disease and MIS-C, preferentially affects children which share an ethnic background shown to be one of the factors predisposing for severe outcome of COVID-19 infection in adults. The cohort study in the UK, investigating the association between patient individual factors and risk for COVID-19 hospital death, revealed as the main risk factors in adults the male gender, advanced age, obesity, and ethnicity (substantially higher risk for people of Asian and African origin) (16).

It is not known why SARS-CoV-2 infection in children overall appears to be mild; however, recent data suggest that children younger than 10 to 14 years are less susceptible to infection of SARS-CoV-2 than adults, while adolescents appeared to have similar susceptibility to adults (19). Although the frequency of asymptomatic SARS-CoV-2 infections among children is unknown, it is possible that the role of children as drivers of pathogen transmission is real (20).

Despite the fact that the proportion of COVID-19 cases that are pediatric has risen substantially from 3% of the reported cases in April to 12% in August and 15% in September in USA (while testing children remained at the same level since April 2020), hospitalization and death due to COVID-19 is still rare (21). Children made up 0.07% of total deaths, and these rates remained stable across the study period (21).

Differences in activity of immune function between children and adults may explain their protection from development of the life-threatening pneumonia. Children have physiologically elevated lymphocyte number, which persist under SARS-Cov-2 infection (22). In addition, children display a reduced production of pro-inflammatory cytokines, such as IL-6 (the main cytokine involved in cytokine storm induction) and a higher production of anti-inflammatory IL-10, as compared to adults upon immune activation (23). Because of that, children may be less prone to develop ARDS. Frequent viral infections during childhood and the scheduled active vaccinations may constantly trigger their innate and adaptive immune system into a state of activation and thus more effectively fight subsequent infections (24). Importantly, over 75% of children seroconvert in response to seasonal coronaviruses before their fourth birthday (20). Although cross-reactivity of antibodies against seasonal coronaviruses and SARS is restricted, it helps to fight SARS-CoV-2 infection in children and young people (20).

However, none of those explanations convincingly elucidate the riddle of the mild COVID-19 presentation in children. Since even infants of affected mothers are protected from the development of COVID-19, we started to believe that an adaptive immune mechanism beyond IgG, IgA, and cytotoxic CD8^+^ T cells is responsible. In order to elucidate this mechanism, we first need to understand the cell biology of coronavirus infection.

## Cell Biology Of Sars-Cov-2 Infection

Coronaviruses (Covs) are enveloped viruses with a positive sense, single-stranded RNA genome (25). Three of them (SARS-CoV, MERS-CoV, and SARS-CoV-2) are known to induce zoonotic disease in men (26). The viral genome encodes four major structural proteins: the spike (S) protein, nucleocapsid (N) protein, membrane (M) protein, and the envelope (E) protein, all required to produce structurally complete viral particles. Within S protein, there is a receptor-binding domain (RBD), which binds to host target receptors, allowing the virus to entry into the host. Previous investigations on SARS-CoV revealed that the virus targets airway and alveolar epithelial cells, vascular endothelial cells and alveolar macrophages for the initial entry into host cells (27).

Coronaviruses are different from other well-studied enveloped viruses in that they bud into the endoplasmic reticulum Golgi intermediate compartment (ERGIC), from where they acquire their membrane envelope (25). Although, the endoplasmic reticulum (ER) is able to degrade misfolded protein in a process called ER-associated degradation (ERAD), if the capacity of ERAD is exceeded, those misfolded proteins trigger an ER stress- and unfolded-protein response (UPR). Further prolongation of UPR results in initiation of apoptosis. Viral infections can also trigger the UPR, adopted by host cells as antiviral strategy (28). Interestingly, CoV E protein exert anti-apoptotic function in infected cells by suppressing the UPR during infection in order to continue viral propagation (25).

The origin of viral envelope from host’s ER in conjunction with the ER stress-response, prompted us to compare between the classical self-nonself and an alternative model of antigen recognition, based on danger signals.

## Limitations Of The Classical View On Adaptive Immunity

Glycosylation is one of the most important post-translational modification of proteins and is characteristic for eukaryotic organisms. Viruses often utilize the host glycosylation machinery during replication and assembly in the host cells (29). Glycans present an overall image of “self” to the immune surveillance of the host organism and may act as protective shell for viral peptide epitopes (30). SARS-CoV does not seem to utilize such a mechanism in order to escape from neutralizing immunoglobulins, like glycan shielding of the receptor-binding site. Instead, a recombinant spike protein fragment with RBD of SARS-CoV presented the highest immunogenicity among other recombinant spike protein fragments tested (31, 32). If SARS-CoV-2 would have similar properties like investigated SARS-CoV, it would help for the successful development of neutralizing monoclonal Abs as therapy. However, alterations in spike proteins, which are emerging as virus spreads and mutates, may render SARS-CoV-2 resistant to monoclonal Abs. Even more surprising is the report that patients who died of SARS-CoV infection achieved their peak levels of neutralizing Abs activity earlier (at 15 days post infection) as compared with those who survived (at 20 days post infection) (33). Adding to this contradiction is the observation that 80% of those patients who progress to ARDS exhibit a successful seroconversion to antiviral IgG (34). Atyeo et al. have correlated disease outcome with the level of specific viral antibodies in patient serum (35). Hereby, they reported that a humoral response specific for viral spike-protein was characteristic for convalescent individuals, while antibodies against nucleocapsid proteins were elevated in deceased individuals. Similarly, children with SARS-CoV-2–associated MIS-C are spared from severe COVID-19 turnover and produce specific IgG antibodies for spike-protein but not against the nucleocapsid-protein (36).

## The Putative Role Of Natural Igm In Sars-Cov-2 Clearance

**Figure 1:**
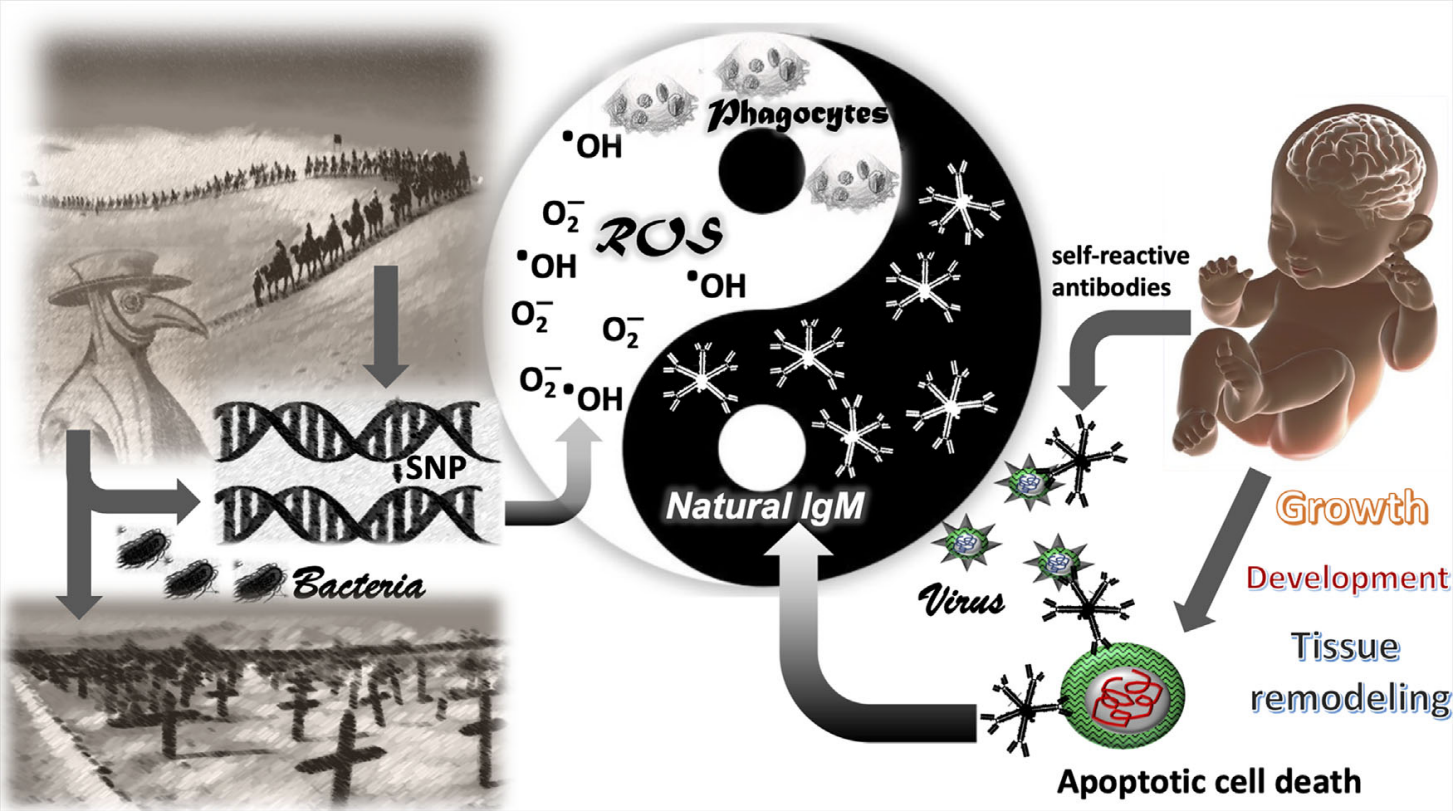
The Yang-Yin of immune defense. The genetic traits favoring the defense against bacterial pathogens in the past (ROS and IgM) appeared to be vulnerable for COVID-19–related mortality in the 21th century. The left side demonstrates how bacterial pathogens shaped the genome in human populations by selective survival of individuals with single nucleotide polymorphisms (SNPs) in genes facilitating bacterial defense, such as the generation of reactive oxygen species (ROS) in phagocytes. The right side shows a newborn with the inherent high polyclonality of its natural IgM. Natural IgM recognize altered self-antigens and are important for removal of apoptotic cells in the developing neonate. Altered self-antigens are also present in the viral envelope of corona viridae. Such as natural IgM removes apoptotic cells in absence of inflammation, the virus is neutralized without inflammatory symptoms and disease. Reactive oxygen species represent the inflammatory and anti-bacterial part of immune defense, associated with Yang in traditional Chinese medicine (TCM). The natural IgM represents the anti-inflammatory and self-recognizing part of immune system involved in tissue remodeling, associated with Yin in TCM.

Due to the fact that young children and even neonates are protected from development of COVID-19, and in view of their naivety in terms of adaptive immunity, such as memory cells and IgG production, we are considering IgM as possible factor responsible for the protection. In particular, natural IgM may play the key role in defense against SARS-CoV-2. Natural IgM constitutes the most abundant immunoglobulin in neonates and exhibits a high potential for self-antigen recognition. The ability to recognize self- or altered self-antigens by the adaptive arm of the immune system is the basic principle of the “danger hypothesis” postulated by Polly Matzinger (37). Consequently, SARS-CoV-2 particles budding from the infected cell are wrapped by the host`s membrane previously modified in the UPR process and thus, are recognized by natural IgM as “altered self”. The same way as apoptotic debris or exosomes are recognized by natural IgM and subsequently prepared for phagocytosis, SARS-CoV-2 as viral particle or as infected cell could be recognized and effectively cleared **(** **Figure 1**).

In the next sections, we will review on natural IgM and their corresponding B1 B cells, summarize their known functions in viral defense, and discuss their putative role in protection against SARS-CoV-2 fatal infection.

## B1 B Cells In Viral Defense

B1 B cells are generated earlier in ontogeny than conventional B cells, namely B2 B cells. The human analog to murine B1 cells is a CD45^+^CD27^+^CD70^-^ B cells subset, reactive with non-protein and self-antigens, such as phosphorylcholine (PC) and dsDNA and detected both in human umbilical cord blood and in the adult peripheral immune system (38). B1 B cells are vastly generated before birth in the fetal liver, are present at high percentage (70–90% of B cells are CD5^+^) in early childhood, drop to a percentage below 10% of all peripheral B cells during adulthood, and further decrease in numbers with age (39).

B1 cells are long-lived and self-renewing and produce more than 90% of the natural antibodies (NAbs), thus providing an important link between innate and adaptive immune systems (40, 41). A key property of NAbs is to maintain immune homeostasis by the clearance of apoptotic debris, the suppression of autoimmune and inflammatory responses and the regulation of B cell development (41). They form the first line of defense against micro-organisms by recognition of structural conserved antigens (both self and non-self).

Since the first study in 1999, aiming to identify natural antibodies specific for viral antigens, many studies have confirmed a role of natural IgM in the protection against viral infections (42, 43). The presence of early neutralizing antibodies during infections with several cytopathic viruses (such as polio, influenza, and rabies) is essential for protection against lethal disease, which often correlates with viral replication in neural tissues (43). As B1 cells arise from fetal liver, they are functionally active in newborns and are crucial for immune protection throughout the time of adaptive immune maturation (41) as IgM production by B1 cells is independent of T cell activation (44). It seems that very early B cells are responsible for the first protection of the newborn organism and that this defense is basically composed of polyreactive IgM (45). Because IgM is not able to cross the placental barrier, the presence of respiratory syncytial virus (RSV)-IgM may reflect the existence of broadly reactive NAbs in newborns (46).

We assume that a similar mechanism in viral immune defense will apply to SARS-Cov-2 in neonates. In contrast to MERS-Cov and the previous SARS-CoV, pregnant women infected with SARS-Cov-2 do not seem to be prone to unfavorable pregnancy outcome (47). Furthermore, SARS-CoV-2 was not detected in products of conception, breast milk, or in neonatal nasopharyngeal swab samples at birth (48). Among mothers with confirmed COVID-19, the virus was not detected in the serum or throat swab of their newborns (48, 49). Interestingly, SARS-CoV-2 IgG concentrations were elevated in five of six infants while increased SARS-CoV-2 IgM levels were observed in two infants (49). Although none of the infants presented any symptoms of COVID-19, significant increase of inflammatory cytokine IL-6 together with increased Ab levels support the possibility of vertical transmission (49). In contrast to IgG Ab, which is passively transmitted from mother to the fetus through the placenta, IgM is not transferred due to its large macromolecular size. While the elevated IgG level may reflect maternal or infant infection, we believe that the increased level of IgM Abs in those neonates suggests that IgM was produced by the infant after the virus crossed the placenta similarly to RSV infection.

Thus, the presence of IgM antibodies in neonates, even in the absence of an elevation, may be sufficient to protect them against COVID-19, when SARS-Cov-2 is present in their mother. The clonal repertoire of B cells producing NAbs is likely shaped by the early life antigenic history of an individual, as neonatal exposure to bacterial antigens result in striking changes in composition of antigen specific NAbs. Thus NAbs reactivity and its potential for antiviral activity is closely related to the species of the viral origin, which is especially important in zoonotic viral infections (29).

## The Natural Igm Antibody (Ab) Repertoire And Its Impact On Defense Against SARS-Cov-2 Infection

Natural antibodies are mostly of IgM isotype, followed by a much smaller fractions of IgG, IgA or IgE isotypes (41). Polyreactive NAbs recognize and bind phylogenetically conserved structures like phospholipids, oxidized lipids, glycolipids, and glycoproteins (41, 50). Current evidence suggests that the pool of B1 cells secreting IgM results from positive selection upon recognition of self-antigens during development. Several IgM and IgA NAbs, recognizing autoantigens, such as phosphatidylcholine determinants in oxidized lipid layers on apoptotic cells, were found binding to homologous molecules in the cell wall of pneumococci (38). Thus, it is likely that composition of the NAbs repertoire is modulated since early childhood by continuous interactions with the microbiome, which is the community of microbial commensals present in our bodies. The cross-reactivity of NAbs between self and microbial antigens also suggests, that vaccination may enhance Ab production and modulate their specificity. Indeed, the protective level of cross-reactive antibodies against oxidized low-density lipoprotein (anti-oxLDL Ab) appeared to be significantly higher after pneumococcal vaccination (51). Similar mechanism may support observations that Calmette-Guérin (BCG) vaccination against tuberculosis is negatively associated with prevalence and mortality of COVID-19 (52).

Several distinct properties of IgM, such as its antigen-binding polyreactivity, the high avidity assured by the ten binding sites, the ability to access cryptic antigens hidden for IgG, and its capacity to promote the removal of apoptotic cells support the specific role of IgM in health and disease (42). Those unique properties allow the recognition and removal of “altered” self-antigens, an ability which is linked to the binding of shared molecules on pathogens, thereby contributing to immune homeostasis controlling inflammation and tissue damage. Indeed, there are several known autoantigens exposed on cells during the process of apoptotic death, which are recognized by subsets of natural IgM (38). Interestingly, IgM that specifically recognize oxidation-associated determinants exposed on apoptotic cells are highly abundant in human newborns (53, 54). Having in mind that SARS-CoV-2 particles leaving the infected cell are covered by the host’s membrane previously modified in the UPR process and thus resembling modifications in apoptotic cells, it is very probable that those particles might be recognized by natural IgM as “altered self”. The same way as apoptotic debris or modified self-particles are recognized by natural IgM and subsequently prepared for phagocytosis, SARS-CoV-2 as viral particle or as infected cell could be recognized and cleared by IgM, highly represented in children’s immune system.

Both natural and induced IgM are polymeric (pentameric) and due to its large size of 970 kDa, IgM does not traverse easily between vascular and extravascular compartments, and therefore, demonstrate specific retention at sites of production or transportation by poly-Ig receptors, such as the mucosal surface, breast milk, or serum (55). That allows natural and induced IgM to act as an early defense mechanism against not only systemic but also mucosal pathogens (56). Those antibodies are conserved in humans and recognize specialized oligosaccharides integrated into the bacterial cell wall and capsule (57). Interestingly, both germ-free and normal conventional-housed mice have similar serum IgM levels, which support the concept that serum IgM concentrations are independent from foreign antigen exposure (42, 58). Additionally, female mice show significantly higher level of serum IgM than male mice (57). The presence of higher levels of oligosaccharide-specific serum IgM results in selective survival of female mice and their offspring, compared to males during bacterial infections. IgM secreting cells seem to be regulated by estrogens, and estrogen-driven antibodies are maternally transferrable to offspring and conferred protection during infancy (57). That might be a reason for the lower fatality rate of severe COVID-19 in females as well as lower incidence of more severe COVID-related diseases in girls in comparison to boys (10, 59).

## Mortality: why some patients end up on ventilators and cytokine storm in covid-19?

The respiratory system is most of all organs involved in continuous environmental exchange and exposed to microbial organisms, due to its large alveolar and mucosal surface, and due to the liters of air moving with every breath. Its essential role in gas exchange and oxygen delivery made it necessary for the lung to evolve strategies to control the inflammation accompanied by antimicrobial defense. Multiple receptors expressed on epithelial and mucosal immune cells directly interact with microbial pathogens, in order to orchestrate the balance between immune defense and inflammation. One of those receptors early associated with SARS-CoV infection is ACE-2, a type I membrane protein proven to be the functional receptor of SARS-CoV and SARS-CoV-2 in the lung (60). ACE-2 is a component of the renin-angiotensin-aldosterone system (RAAS), which represents a cascade of vasoactive peptides, controlling main processes in human physiology like blood pressure, wound healing, and inflammation (61). The key role of ACE-2 is to covert angiotensin II (AngII) into angiotensin-(1-7), which was shown to have anti-oxidant and anti-inflammatory (including antithrombotic and anti-fibrotic) effects (62, 63).

Although the role of ACE-2 in SARS-CoV-2 infection is conflicting, there is increasing evidence in published literature that a high level of ACE-2 expression is rather beneficial than harmful in patients with lung injury (61). ACE-2 expression is high in infants, reaching a plateau in adolescence, and decrease during adulthood, in males faster than in females (64). It was speculated, that the decline in sex hormones contribute to the observed decrease in expression of ACE-2 with age, as estrogen and androgens activate ACE-2 expression (64). The analysis of ACE-2 expression across age, gender, and ethnicity showed that ACE-2 expression level is high in Asian females and young people, suggesting a negative correlation between ACE-2 expression and SARS-CoV-2 severe outcome (64). Compared to children, older patients present reduced the number of ACE-2–expressing cells and lung progenitor cells, which made those patients more vulnerable to develop severe pneumonia with poor recovery potential from COVID-19 (65).

Thus, children having high expression of ACE-2 seem to be protected against angiotensin II accumulation and its harmful cytotoxic effects and benefit from the anti-inflammatory properties exerted by angiotensin-(1-7).

Another receptor playing a key role in pulmonary viral defense and inflammation is retinoic acid-inducible gene (RIG)-I which recognize RNA of a variety of virus families (66). Upon viral RNA recognition, RIG-I together with adapter molecule mitochondrial antiviral-signaling (MAVS) protein triggers a cascade leading to the activation of the inflammasome, culminating in the expression of antiviral interferons (67). Most recent evidence suggests that overactivation of the inflammasome by SARS-CoV-2 is the responsible factor for the cytokine storm (68). Interestingly, inflammasome and certain adaptor molecules for RIG-I signaling are located at the mitochondrial outer membrane (67). Mitochondria serve a key role in the supply of Krebs cycle and lipid metabolites and are the main responsible organelles for maintenance of the cellular redox equilibrium. The level of inflammasome activation *via* RIG-I and viral RNA is dependent on ROS (69) through upregulation of the mitochondria-associated adapter MAVS (70). The activity of complex multiunit enzymes belonging to the NADPH oxidase (NOX)- and the dual oxidase (DUOX) families, both expressed in airway- and alveolar epithelial cells, is catalyzing the local generation of ROS subsequent to viral challenges (71).

In this review, we hypothesize that a massive increase in production of ROS triggered by assisted ventilation under high oxygen pressure and facilitated by the downregulation of ACE-2 and the viral load leads to a vicious cycle between RIG-I signaling, exacerbated inflammasome activation and ROS production, ending up in a cytokine storm. There is accumulating evidence that the assisted ventilation in patients with COVID-19 does not change the disease course (2, 72). In addition, iron as limiting element for the continuous activity of NOX and DUOX is reduced in its availability by the upregulation of ferritin, in order to avoid accumulation of ROS and thereof cellular toxicity. Indeed, patients presenting a severe form of COVID exhibit high levels of serum ferritin (73–75), suggesting an inflammatory process accompanied by high levels of ROS.

## Genetics of ROS Generation and Human Sociographic and Linguistic Evolution

The key regulatory checkpoints in ROS production are determined by activity and localization of the multiunit enzymes NOX and DUOX. While epithelial cells of the upper respiratory tract express several isoforms of NOX and DUOX, alveolar epithelial cells, macrophages, and vascular endothelial cells express only two isoforms of NOX, namely NOX2 and NOX4 (76). Macrophages and granulocytes require NOX2 for generation of sufficient ROS in lysosomal compartments for the elimination of bacterial pathogens. The significance of NOX2 function for bacterial defense is apparent in patients with chronic granulomatous disease (CGD), where several identified mutations in any of the five subunits of NOX2 lead to high susceptibility for bacterial infection, giving rise to a low life expectancy (77). Since the first description of a genetic mutation in a NOX subunit as cause for CGD in the 1980s, single nucleotide polymorphisms (SNPs) in several NOX subunits and in enzymes involved in neutralization of ROS were identified and associated with atherosclerosis (78), type II diabetes mellitus (79), diabetic nephropathy (79), and thrombosis (80). However, besides the relevant SNPs, the genetic background of patients in those studies, as reflected by their ethnicity, seems to have a significant impact on the outcome. Interestingly, the small p22(phox) subunit, shared between NOX 1- 4 enzymes, serves a crucial role in the assembly and intracellular localization of all other enzymatic subunits and was found to be highly polymorphic with functional differences in ROS generation (81).

Consistent with the role of ACE-2 in neutralizing ROS and decreasing angiotensin II, there is evidence that a persistent high activity of angiotensin II is at least in part responsible for the organ injury observed in COVID-19 (82, 83). SARS-CoV-2 downregulates ACE-2 expression after using it for cellular entry, resulting in unopposed angiotensin II accumulation and local RAAS activation (84, 85). The level of plasma angiotensin II correlates with the degree of lung injury and total viral load in COVID-19 patients (83).

Before the invention and wide applications of antibiotics in the 1950s, bacterial infection represented the major cause of death in the human population (86). Bacteria and its ability for host adaption has shaped human evolution for thousands of years by maintaining selective pressure on immune function in young individuals before attaining their reproductive age. Polymorphisms in genes associated with functional differences in generation or neutralization of ROS, or its subcellular localization in phagocytes, may had a profound impact on survival and subsequent genetic inheritance during the pre-antibiotic times (**Figure 1**). In human history, populations migrated and settled in regions supporting and improving their livelihood. Ancient trade routes, such as the silk route, provided an economic belt for settlement, connecting Southeast Asia with Southern Europe and spanning a period of 1,500 years. Along this route, the movement of people with diverse ethnic and genetic background was accompanied by rodents, parasites, and pathogens. Thus, ancient trade routes, such as the silk route, may have generated and selected genetic variants for bacterial immune defense, facilitated by merging the genetic background of multiple ethnic groups under the challenge of an aggravated bacterial exposition. Until today, the prevalence of Behcet’s disease, an autoimmune vasculitis of unknown etiology, is located along the countries of silk road (87), suggesting a selection process for distinct immunological traits along this route. Remarkably, a predisposition for distinct symptoms, such as vasculitis and thrombosis, is shared between Behcet’s disease and severe COVID-19. Interestingly, mutation of GM-CSF—one of the major cytokine initiating and perpetuating inflammatory diseases was shown to be also one of the risk alleles in developing Behcet-like disease (88). Thrombosis is frequently observed in COVID-19 patients suffering an aggravated disease course (89), and vasculitis resembling Kawasaki disease was specifically observed in children, subsequently to a suspected contact with SARS-CoV-2 (90). When connecting countries and regions with high mortality due to COVID-19 or persistent high infection rate of SARS-CoV-2 on a map in the early 2020, those countries are part of the former silk road, such as Iran, Turkey, northern Italy and southern China. Wuhan, the epicenter of COVID-19 is in less than 1,000 km distance from Guangzhou, the beginning of the ancient silk road, both cities were haunted by an extraordinary high rate of mortality during the epidemics (2, 91). On the other end, France and south of Switzerland sharing linguistic, cultural, and economic connections to northern Italy over a long-lasting period in human history. Both regions were heavily affected by COVID-19 related mortality (92). A similar indication is found in Canada, where exclusively the French-speaking Quebec is severely affected by COVID-19 mortality.

Thus, we propose the hypothesis, that during ancient times, over a period of 1,500 years along the silk road, distinct genetic traits affecting the redox equilibrium were shaped in the local inhabitants. As much those genetic traits favored the defense against bacterial pathogens in the past by an appropriate production of bactericidal ROS, the same genetic traits prove them vulnerable for COVID-19 related mortality in the 21^st^ century as presented in **Figure 1**.

## Discussion

The pathology of COVID-19 extends far beyond a pulmonary disease and does not resemble any other viral pneumonia reported so far (23). In order to search for therapeutic approaches, COVID-19 needs to be viewed as complex interaction between pulmonary, hematologic, and endothelial dysfunction in the context of an inflammatory condition. To solve such an intertwined mechanism, it is of great value that medical scientists investigate children and young adults due to their vastly different clinical outcomes.

Although a vast amount of clinical data and results in the basic science on COVID-19 are published up to date, the mechanism of severe disease progression, the differential in the rates of mortality depending on geographic location or ethnicity, and the reason why children and young adults are mostly protected from development of disease are unknown. In this review, we performed an approach to answer those questions by integrating historical circumstances, current clinical data and little noticed elements of immunity. Hereby, we propose that infants, children and young adults are protected from SARS-CoV-2 due to the polyclonality of natural IgM, able to recognize viral particles or infected cells by endogenous self-antigens, according to the danger model of immunity. The differences in severity of COVID-19 observed in adults, may have their roots in the genetics of bacterial defense mechanisms, revealing themselves as harmful in COVID-19 when combined with mechanical ventilation under high oxygen pressure.

In order to test our hypotheses, we propose the genetic analysis of children with Kawasaki-like disease and MIS-C with attention to genes involved in IgM Ab response and reactive oxygen metabolism. Furthermore, retrospective analysis and prospective studies on the use of nutritional supplements with antioxidant properties, such as vitamin C, are required to assess their potential impact on disease outcome in COVID-19. The search for correlations between the severity of SARS-CoV-2 infection and single nucleotide polymorphisms in genes involved in ROS metabolism would test our hypothesis.

Recent clinical observations support our hypothesis. Although it is currently uncertain whether immunomodulating agents, such as biological disease-modifying anti-rheumatic drugs (bDMARDs), affect the outcome of SARS-CoV-2 infections, the individual cases reported that bDMARDs treatment does not correlate with worse COVID-19 outcome (93). Also according to the guidelines of European League Against Rheumatism (EULAR), the immunomodulatory treatment advised to rheumatic patients should be continued (94). However, due to recently reported two fatal cases, some caution should be applied when using rituximab (RTX), a B-cell depleting bDMARD in patients with immune-mediated diseases (95). This observation is supported by a report on persistent SARS-CoV-2 viremia in two rituximab-treated patients with severe COVID-19 pneumonia and death without any sign of viral clearance (96). Alarming data from the German National Registry for patients with IRD infected with SARS-CoV-2 show that 61% patients treated with RTX required hospitalization while 50% needed ventilation (97). Indeed, the consequence of the RTX administration is very low IgM concentration, which may support our hypothesis of an important role of IgM in protective immunity against COVID-19 (98). Additionally, the mild clinical course of COVID-19 in patients with agammaglobulinemia suggest that it is not IgG that protect from severe or fatal COVID-19 course (99).

## Conclusions And Suggestions For Potential Therapeutic Interventions

Concluding from our hypothesis on reactive oxygen species as key factor for COVID-19 related mortality, we suggest the use of an amphiphilic antioxidant for targeting ROS in lysosomal compartments. The mild sedative drug melatonin shares antioxidant and amphiphilic properties, along with a low potential for adverse effects (100). Another alternative to COVID-19 treatment due to its antioxidant activity and cytoprotective effects may be the most abundant free amino acid in humans, taurine (101). Taurine regulates inflammatory processes associated with oxidative stress, such as the detoxification of hypochlorous acid, which results in formation of less toxic and anti-inflammatory taurine chloramine. We suggest the identification of polymorphisms in genes related to reactive oxygen metabolism to estimate the potential susceptibility of infected patients for severe disease progression and clinical trials with intravenous immunoglobulin preparations, enriched for IgM in the early stage of disease.

## Data Availability Statement

The original contributions presented in the study are included in the article/supplementary material. Further inquiries can be directed to the corresponding author.

## Author Contributions

MM and H-JG wrote the manuscript. H-JG revised the manuscript for important intellectual content. All authors contributed to the article and approved the submitted version.

## Funding

This work was supported by core grant S/9 to the National Institute of Geriatrics, Rheumatology, and Rehabilitation from Polish Ministry of Science and Higher Education.

## Conflict of Interest

The authors declare that the research was conducted in the absence of any commercial or financial relationships that could be construed as a potential conflict of interest.
